# Automated Analysis of Barley Organs Using 3D Laser Scanning: An Approach for High Throughput Phenotyping

**DOI:** 10.3390/s140712670

**Published:** 2014-07-15

**Authors:** Stefan Paulus, Jan Dupuis, Sebastian Riedel, Heiner Kuhlmann

**Affiliations:** Institute of Geodesy and Geoinformation, Department of Geodesy, University of Bonn, Nussallee 17, 53315 Bonn, Germany; E-Mails: j.dupuis@igg.uni-bonn.de (J.D.); sr_sebi@web.de (S.R.); heiner.kuhlmann@igg.uni-bonn.de (H.K.)

**Keywords:** laser scanning, automatic parameterization, barley, automatic classification, plant phenotyping

## Abstract

Due to the rise of laser scanning the 3D geometry of plant architecture is easy to acquire. Nevertheless, an automated interpretation and, finally, the segmentation into functional groups are still difficult to achieve. Two barley plants were scanned in a time course, and the organs were separated by applying a histogram-based classification algorithm. The leaf organs were represented by meshing algorithms, while the stem organs were parameterized by a least-squares cylinder approximation. We introduced surface feature histograms with an accuracy of 96% for the separation of the barley organs, leaf and stem. This enables growth monitoring in a time course for barley plants. Its reliability was demonstrated by a comparison with manually fitted parameters with a correlation *R*^2^ = 0.99 for the leaf area and *R*^2^ = 0.98 for the cumulated stem height. A proof of concept has been given for its applicability for the detection of water stress in barley, where the extension growth of an irrigated and a non-irrigated plant has been monitored.

## 1. Introduction

Measuring the 3D plant architecture is a common and well-integrated method in plant biology and plant breeding [1,2]. Parameters of the plant architecture can be acquired [3], as well as organ-based parameters [4]. Applications have been shown at the field [5], greenhouse [6] and laboratory scale [7].

Linking the genetic information to the geometric appearance, which is influenced by the environmental parameters, has become a strong focus in plant science [8]. One key aspect is the non-invasive tracking of extension growth. Focusing on in-depth phenotyping, the exact shape description of plants is essential to monitor growth or reaction due to environmental changes [8,9]. Highly resolved and highly accurate point clouds enable the detection of the smallest changes and subtle deformations. Laser scanning is a common technique in plant science, which can satisfy demands regarding resolution, accuracy and completeness [1,2,10]. Current applications have demonstrated its applicability for the extraction of the root volume of trees [10], biomass [11] or the prediction of yield parameters [12].

A common way to measure extension growth is the parameterization of the plant organs and tracking these parameters over time [13]. The current literature [7,14] defines a gap for a barley experiment, where the laser scanned point clouds have to be manually separated to the single organs. This needs either intensive preprocessing [15] or demands a great deal of work. One approach focuses on the automated separation of plant organs due to their surface geometry [12]. This had been proven for the separation of stem and leaf points of grapevine, as well as for the separation of wheat ear and stem. Nevertheless, its application must be adapted for every plant type. This has, so far, not been tested for the parameterization of barley leaves and stems and for the application in a time course, in particular.

The parameterization of plant organs is possible, if the single organs can be clearly identified. For each organ type, a specific parameterization has to be used. Leaves were commonly depicted as triangle meshes, which provides highly accurate results [7]. For the stem organs, the representation by cylinder primitives is a common technique [14,16]. Parameters, like stem height, perimeter or volume, can easily be derived from this representation and can be used, e.g., for plant modeling [14,17] or to measure the growth response on nitrogen fertilizer at different levels [18]. The automatization of the cylinder estimation is a key aspect for the application in high-throughput phenotyping.

High-throughput phenotyping focuses on the measuring of thousands of plants during many measuring dates and with many repetitions [8]. The more crosses and environments that were used for phenotyping, the greater the probability of identifying superior variations [19]. These datasets need an automated analysis to prevent labor and time-intensive manual work [20], like the separation of the organs and their parameterization [7].

A clear parameterization enables the generation of functional-structural plant models [21]. These models are able to encode geometry (shape) and functional processes [22], enable growth modeling due to, e.g., drought stress [23], allow the prediction of geometrical deformation, model the signal propagation within the plant [24] and provide the simulation of the light distribution within a canopy [25]. The model-generation process can be accelerated, when algorithms for the transformation of the 3D data into a parameterized connected structure were available.

A prerequisite for that and the goal of tracking growth is the ability to satisfy the demands of phenotyping regarding automatization and high throughput. The aim of this study was to show the applicability of surface feature histograms for use on laser scanned barley point clouds to identify plant organs and to provide an accurate parameterization for the organs, leaf and stem. The advantages of this parameterization are shown in a barley experiment, where the parameter of an irrigated and a non-irrigated plant were evaluated by manually-fitted cylinders for the stems and triangle meshes for the leaf organs. This approach shows how the complete above-ground plant structure of leaves and stems can be parameterized automatically to monitor plant extension growth in a time course.

## 2. Method

### 2.1. Workflow and Experimental Setup

The shown approach combines the identification of organs of a barley plant scan using a surface feature histogram-based classification technique with a mathematical description of the morphological organs to enable extension growth monitoring over a period of 21 days.

**Figure 1.**
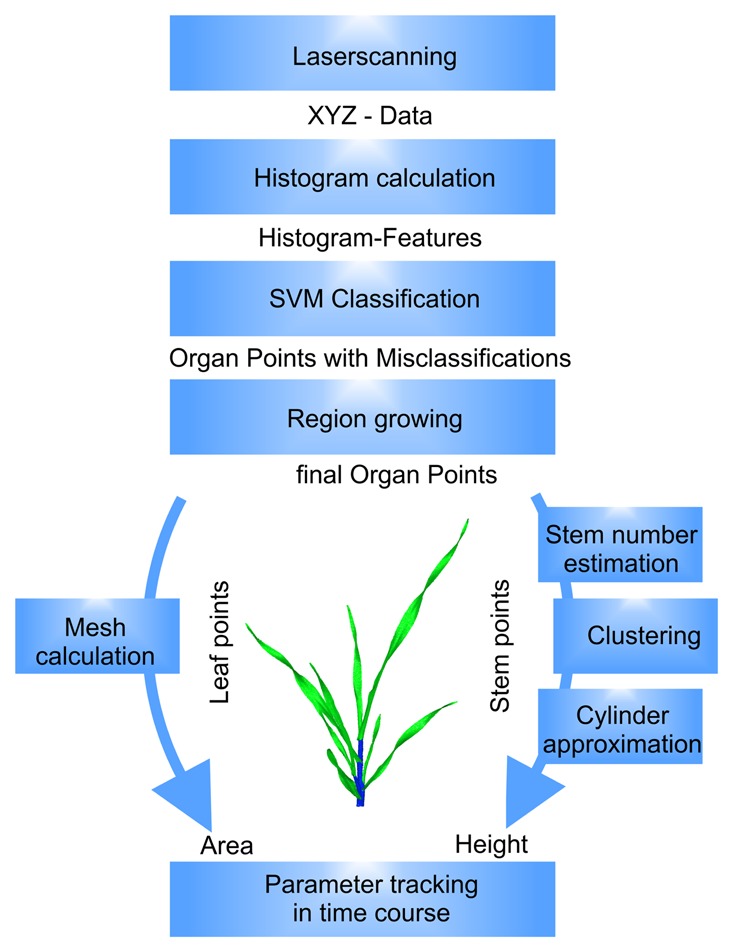
Data flow for the parameterization of the barley plant point cloud. The laser scanner provided XYZ point clouds that were classified, refined by a region growing algorithm and parameterized due to their organ type. This enabled parameter tracking in a time course.

To give a short overview of the method, Figure 1 shows the data flow together with the processing steps from the input of laser scanner data to the calculation of surface feature histograms and the organ identification using support vector machines (SVM [26]) and region growing. As relevant plant organs, the leaf, blade and sheath, as part of a phytomer, and the stem, as a stack of internodes, were the focus. The leaf properties were represented by the leaf area, which was calculated from a mesh-representation. The stem parameter was calculated by a stem number estimation using an adapted Gauss clustering algorithm, a hierarchical clustering to identify the single stem clusters, followed by a least-squares approximation of a cylinder model.

**Figure 2.**
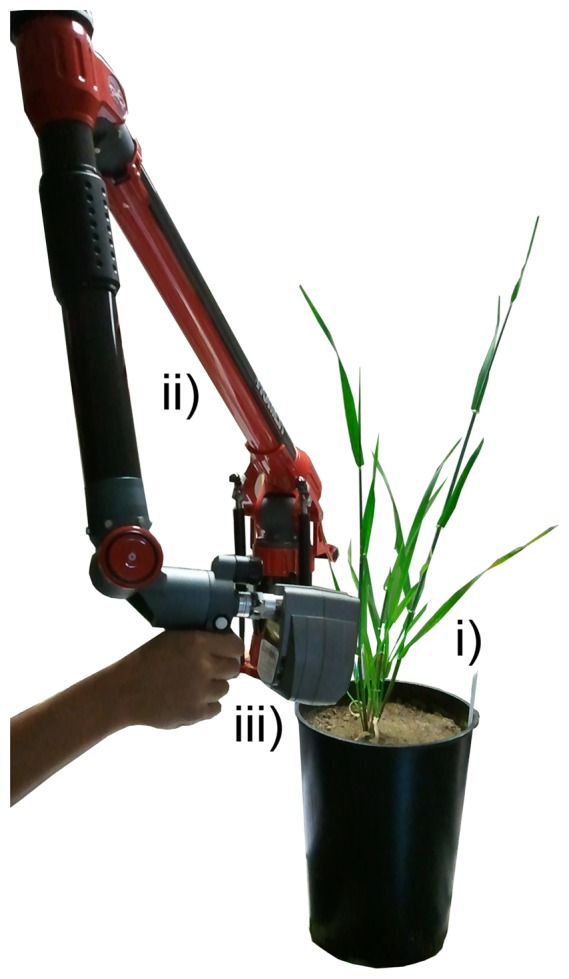
Laser scanning of a barley plant (**i**) using a measuring arm (**ii**) coupled device (**iii**) with a measuring sphere of 1.4 m in radius (Romer Infinite 2.0 and Perceptron V5, Plymouth, MI, USA).

For the data acquisition, a Perceptron V5 laser scanner (Perceptron Scan Works V5, Perceptron Inc., Plymouth, MI, USA) coupled to an articulated measuring arm (Romer Infinite 2.0, Hexagon Metrology Services Ltd., London, UK) was used. This combination offered a spherical measuring volume of 1.4 m in radius and a mean point to point distance of 14 μm [27]. The measurement accuracy was defined by the manufacturer as 45 μm [28]. Figure 2 shows the laser scanning device together with the coupled articulated measuring arm during a barley scanning experiment.

For the plant experiment two spring barley plants (*Hordeum vulgare L.*, CV. Barke) were cultivated in plastic pots (volume = 5.5 L, height = 20 cm, diameter =16 cm) in a substrate mixture of topsoil, sand, peat and milled lava in a foliar tunnel (location: N 50°43′ E 007°05′, University of Bonn, North-Rhine-Westphalia, Germany, April–May 2012). All pots were well watered for germination. The measurements started sixteen days after seeding (BBCH11–31). At this point, the irrigation was stopped for one plant; the other one was watered regularly (the first observation date was called day 0). The measurements were performed in a laboratory on ten measuring dates on days 0, 3, 5, 7, 10, 12, 14, 17, 19 and 21. After this point of time, the scans of the irrigated plants provided many occlusions and missing parts, due to the geometric dimension of the plant and the limited field of view. The laser scanners manual handling led to differences in the density of the point cloud. For this reason, the point cloud was thinned out to a homogeneous resolution with a minimal point-to-point distance of 1 mm using Geomagic Studio 12 64 Bit (Raindrop Geomagic Inc, Morrisville, NC, USA).

### 2.2. Separating the Organs Using Surface Feature Histograms

The laser scanner provided a 3D point cloud using Cartesian coordinates without any semantic information. A histogram-based classification method [12] that was previously applied to laser scanned point clouds of grapevine and wheat was applied to identify the points belonging to the single organs of barley. Surface feature histograms use 3D points and their normal vectors within a small neighborhood to calculate a representation of the surface geometry encoded in a histogram [29].

The normal vector of a source point was calculated from points within a defined radius (*r_N_*) around this point using principal component analysis [30].

**Figure 3.**
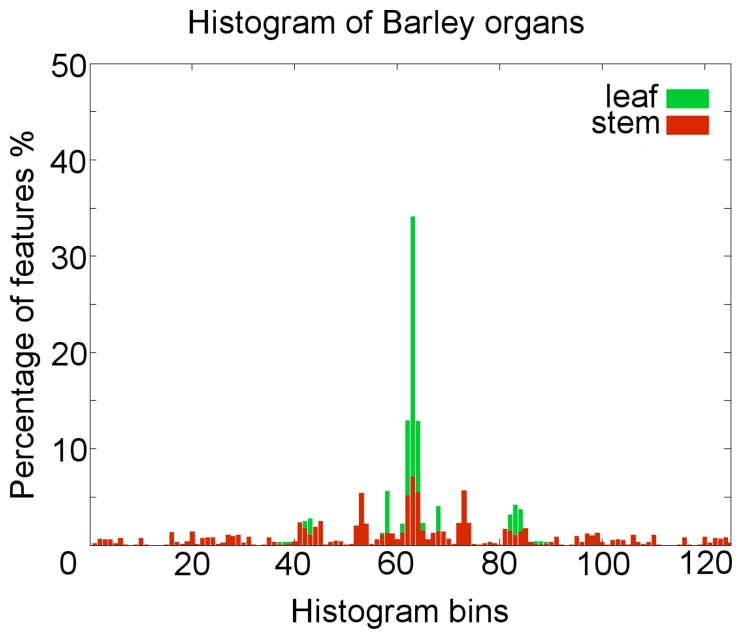
Surface feature histogram representations were calculated for every point in the point cloud. The mean 125-bin histogram is shown for points from the leaf point cloud and the stem point cloud.

To create the histograms representation for a specific point, the azimuthal angle of two neighbor normals, the cosine of the polar angle, as well as a measure for the direction and length of the translation between the two points were calculated. These features have to be calculated between the source point and every neighbor point within the radius *r_H_*. Typical surface feature histogram descriptions for the barley organs are shown in Figure 3.

To achieve the best classification results, the histogram properties had to be adapted for every new classification scenario, *i.e.*, plant type and organ focus. Only a sufficiently small radius *r_N_* enables the representation of detailed surface structures, whereas a radius *r_N_* that is too small is vulnerable to the effects of noise. Nevertheless, the radius *r_H_* should be chosen carefully to avoid a strong smoothing of the histograms on highly structured surfaces. Based on the findings of [12], the support vector machine (SVM) algorithm implemented in libSVM [31] using radial basis kernels [32] was applied.

The parameterization of the leaf area could be conducted directly after the classification step. Therefore, the meshing algorithm, implemented in Geomagic Studio 12, was used. The underlying point cloud resolution of 1 mm provided a smooth leaf surface.

### 2.3. Choosing the Histogram Parameters

Following the parameter extraction pipeline shown in Figure 1, the first step was the identification of the barley organ point clouds using surface feature histograms. The work of [30] defined that these histograms have to be adapted to their application scenario with respect to the point cloud resolution and the object geometry. The two parameters influencing the histogram characteristics were the radii for the normal calculation *r_N_* and for the histogram calculation *r_H_*. Only clear separable histograms enable a high classification accuracy.

In our method, the parameters for the histogram classification were evaluated in an interval between 2 mm (doubled resolution) and 15 mm as the size of the biggest object, the stem. The optimal histogram parameters for barley plants were determined in a cross-validation experiment using the manually-labeled point cloud of the irrigated plant of measuring date 21. This resulted in a fixed radius for all plants *r_N_* = 3 mm and *r_H_* = 15 mm. Bigger radii extend the calculation time, but do not improve the classification result. To face the problem of occurring misclassifications, a region growing algorithm was applied, where smaller regions were connected to the bigger neighbor regions [12].

### 2.4. Clustering the Single Stems

After the identification and parameterization of the leaf points, the points labeled as stem were the focus. For the least-squares estimation of the describing cylinder, the single stems had to be identified. This could be offered by a clustering step. An estimation of the stem number simplified the clustering of the single stems by providing the amount of required clusters and enabled the use of standard clustering routines for a subsequent cylinder estimation. The workflow included: (i) estimation of the stem orientation; (ii) the creation of a Gauss-orientation clustering; (iii) the application of a threshold with a subsequent calculation of the amount of local maxima; and (iv) a final hierarchical clustering of the single stems.

The stem number was estimated by extracting the axis of the stems. A several times-repeated line fit showed the stem axis with a high probability and an accumulation at this specific stem direction, respectively.

**Figure 4.**
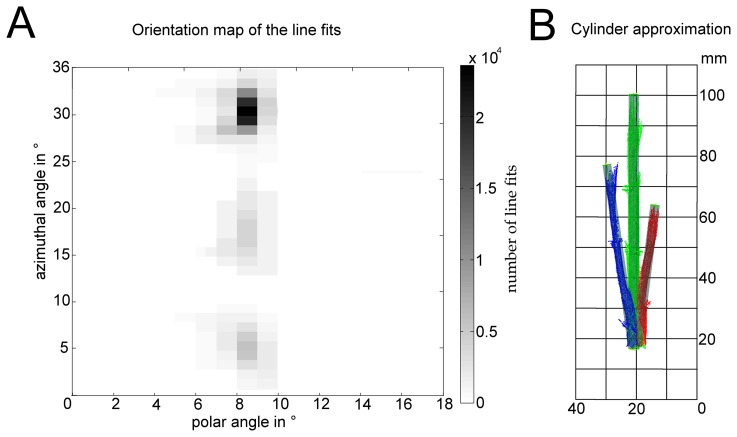
The result of the octant-wise line fits is a heat map showing the summarized direction vectors using a spherical representation showing three local maxima (**A**). The grayscale encodes the amount of lines with the same orientation. For every cluster, a least-squares approximation of a cylinder is calculated (**B**). The first stem is colored green, the second blue and the third is colored red.

Therefore, the plant point cloud center of gravity was moved to the origin of the coordinate system. Empirical tests recommended using a space division into the eight Euclidean octants and performing a 1000-times repeated line fit out of five randomly chosen 3D points for every octant using MATLAB 2009 (The MathWorks Inc., Natick, MA, USA). The stem axis has a high probability of being estimated very often. According to Gauss-clustering [33], the direction vectors (1000 vectors per octant) of the line fits were mapped to a unit sphere according to their spherical coordinates with azimuthal and polar angles. Therefore, the azimuthal angle *ϕ* = [0 … 360°] and the polar angle *ω* = [0 … 180°] were subdivided using 10° steps. By this, we generate a voting for the stem direction, where the stem axis corresponds to the highest votings. The amount of local maxima corresponds to the number of stem cylinders and, furthermore, to the amount of clusters. Using a threshold of 20%, connected regions in a black-white map became visible. Figure 4A shows the results of the spherical mapping of the direction vectors resulting from the line fits using the classification of measuring date 14 of the irrigated plant scan. This representation shows that three stems generate three appropriate clusters with a high voting.

Every region corresponds to the orientation of a single stem. Thus, the amount of regions corresponds to the amount of cylinders within the point cloud. This enabled the use of a hierarchical clustering technique [34]. In detail, an agglomerative method with single linkage and an Euclidean distance measurement resulted in one separated cluster for every stem. A parameter estimation for every stem cluster could now be conducted. Therefore a least-squares method was used to estimate one cylinder for every stem cluster.

Figure 4B shows the result after the least-squares cylinder approximation.

### 2.5. Parameterization of the Stems

Previous steps provided the amount of stems, as well as a cluster allocation. The cluster points were used to estimate the parameters of the related cylinder jacket following a least-squares approximation approach [35]. Therefore we used the following implicit form to parameterize the cylinder (Equation (1)). There, in **Φ**, a point on the main cylinder axis is described by ***P*** = (*x_p_*, *y_p_*, *z_p_*)*^T^* , the orientation as a normalized vector ***a*** = (*x_a_*, *y_a_*, *z_a_*)*^T^* , the radius *r* and the points ***Q*** on the cylinder coat. Equation (1) describes the cylinder in an implicit form using **Φ** to define the functional model.

(1) $$\text{Φ}=\left\|(\text{P}-{\text{Q}}_{\text{i}})\times \text{a}\right\|-r=0$$

‖…‖ denotes the norm of the vectors and × describes the cross-product. Following [36], who used cylinders to describe tree stems, the parameters were reduced by determining the length of ***a*** = 1 and ***P*** lying in a freely selectable plane with *z_p_* = *h*. Using more parameters and defining conditions between these parameters simplified the processing. This leads to the restriction *γ* [37]:

(2) $$\gamma =\left[\begin{array}{c} \gamma_{1}\\ \gamma_{2} \end{array}\right]=\left[\begin{array}{c} \left\|\text{a}\right\|\\ z_{p} \end{array}\right]=\left[\begin{array}{c} 1\\ h \end{array}\right]$$

This approach led to a restricted Gauss–Helmert model. The following equation represents the system of normal equations for the variable *x*, including the parameters ***P***, ***a***, *r*:

(3) $$\left[\begin{array}{c} \hat{\text{x}}-{\text{x}}^{0}\\ k \end{array}\right]=\left[\begin{array}{cc} -{\text{A}}^{\text{T}}{\left({\text{B}}^{\text{T}}\text{QB}\right)}^{-1}\text{A} & {\text{C}}^{\text{T}}\\ \text{C} & 0 \end{array}\right]=\left[\begin{array}{c} {\text{A}}^{\text{T}}{\left({\text{B}}^{\text{T}}\text{QB}\right)}^{-1}{\text{w}}_{1}\\ -{\text{w}}_{2} \end{array}\right]$$

The matrices ***A*** and ***B*** were derived from Equation (2); the matrix ***C*** was derived from the restriction (Equation (3)). *x̂* denotes the solution vector that was updated with every iteration step and k denotes the vector of Lagrange multipliers. Initial starting values (*x*^0^) were given for P as the centroid of the point cloud and for ***a***^0^ = (0, 0, 1)*^T^* as the barley plant stems grow to the top.

Using *v* to mark the residual vector, the matrix ***A***, ***B***, ***C*** can be defined as:

(4) $$\begin{array}{ccc} \text{A}={\frac{\partial \phi (\text{υ},\text{x})}{\partial \text{x}}|}_{{\text{υ}}_{0},{\text{x}}_{0}} & \text{B}={\frac{\partial \phi (\text{υ},\text{x})}{\partial \upsilon }|}_{{\text{υ}}_{0},{\text{x}}_{0}} & \text{C}={\frac{\partial \gamma (\text{x})}{\partial \text{x}}|}_{{\text{x}}_{0}} \end{array}$$

*w*_1_ and *w*_2_ represent the discrepancies as:

(5) $$\begin{array}{cc} {\text{w}}_{1}=-\text{B}{\text{υ}}^{0}+\phi ({\text{υ}}^{0},{\text{x}}^{0}) & {\text{w}}_{2}=\left[\begin{array}{c} \left\|\text{a}\right\|-1\\ z_{p}-h \end{array}\right] \end{array}$$

*υ*^0^ describes the initial residual vector (0, 0, 0)*^T^* that was updated iteratively. The equation system was solved using established procedures [37]. If all points were used, the cylinder approximately would result in a cylinder with a radius that is too big, due to the stem points that dispersed in the upper and lower end of the cluster. To overcome this physiological problem, the least-squares approximation was calculated using only six points. The robustness could be improved by using the RANSAC algorithm [38]. Thereby, the accurate separation of the points belonging to the different stems by the clustering algorithm reduced the required iterations to about 50. Finally, the result with the smallest discrepancy was chosen.

The length of the cylinder was determined using two steps. The estimated cylinder was extended along its main axis (Step 1). As long as a sufficient amount of points of the neighborhood was within a specific range (set to 0.5 mm), this elongation step was repeated. The highest and lowest point of the cylinder coat, projected on the main axis, determine the length of the stem cylinder (Step 2).

All experimentally defined thresholds had been chosen for one test dataset and then kept static for the classification and parameterization of the time series dataset.

### 2.6. Application in a Barley Experiment

The manually-labeled plant of measuring day 21 was used for the training of the SVM model. This was applied to classify the plants of the days 0 to 19 of the irrigated and the days 0 to 21 of the non-irrigated barley plant, respectively.

Resulting in a mathematical description of the barley stems, the size, e.g., height, could easily be derived. The automatically extracted data of two monitored barley plants were compared to the manually extracted reference data using Geomagic Studio 12. For the reference leaf area and the reference stem height, the organs were segmented by hand, and the parameterization for both organs (triangle mesh and cylinder) was calculated with Geomagic Studio routines. For the evaluation, a cumulated approach was exhibited, this could be done fully automatically and did not need a specific alignment of the single stems to monitor extension growth on the organ level.

## 3. Results

To evaluate our method, it was applied to an experiment with two barley plants, scanned on ten measuring dates during a period of 21 days. The histogram representation provided satisfying results and a clearly separable representation for the points of the barley organs, leaf and stem (Figure 3).

**Figure 5.**
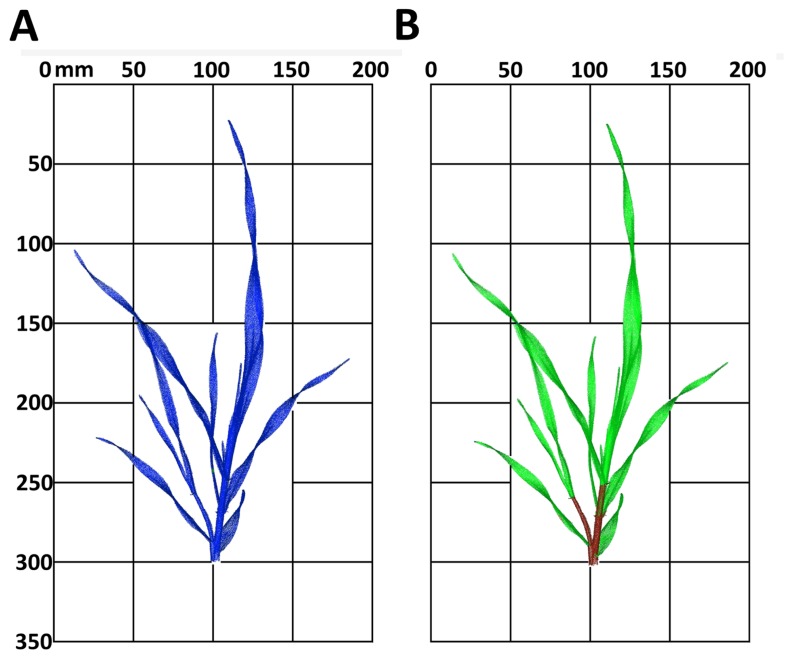
The classification of the raw point cloud (**A**) of the irrigated barley plant from measuring date 17 into the plant organs leaf and stem (**B**) was realized.

Figure 5 shows the appearance of the raw point cloud of the irrigated plant from day 17 (Figure 5A). The classification shows a clear separation of the plant organs with a high accuracy of 95.3% (Figure 5B), calculated by the accuracies of the classes, stem and leaf, weighted with the amount of pixels.

Table 1 shows a detailed view on the size of the single point clouds for both treatments and for each measuring date together with the number of leaves and stems. The accuracies for each classification were calculated using the manually labeled point cloud of measuring date 21 for training. A mean accuracy of 93.97% was reached for the classification of the leaf points and 82.09% for the stem points.

**Figure 6.**
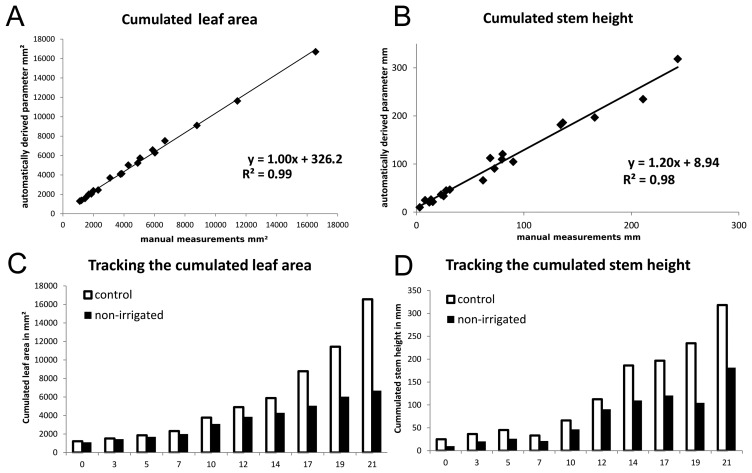
The correlation of the leaf area compared to manually-taken parameters is shown in (**A**); (**B**) the comparison for the parameter cumulated stem height. Repeated measurements of the irrigated and the non-irrigated plant can be used for the tracking of defined parameters in the time course (**C,D**).

To evaluate the reliability of the automatically-derived parameters, they were compared to manually-taken parameters. Figure 6A shows a high correlation of *R*^2^ = 0.99 for the automatically-derived cumulated leaf area compared to the reference measurement. The cumulated leaf area of 20 datasets (two plants of ten measuring dates, irrigated and non-irrigated) was used. A root mean square error (RMSE) of 341 mm^2^ was reached.

In Figure 6B, a similar high correlation of *R*^2^ = 0.98 at an RMSE of 24.13 mm^2^ for the stem height, compared to the reference method, was reached. As before, the cumulated stem height of 20 barley plant datasets was considered.

To show the applicability of the used method for monitoring growth effects due to water supply in the time course, the cumulated leaf area is visualized in Figure 6C. The measurement started on the day when the watering for the non-irrigated plant stopped. A clear growth trend is visible for both treatments, the control and the non-irrigated plants. Nevertheless, a difference between both plants can be clearly observed (Figure 6D).

The differences between the treatments for the cumulated leaf area and the cumulated stem height become visible in the results automatically extracted from the laser scanning data. The difference started for the leaf area from 1 mm^2^ on day 0 and reached 98.8 mm^2^ on day 21. The difference in stem height started on day 0 with 14.9 mm and ended with 136.7 mm on the last measuring day.

## 4. Discussion

Both correlation graphs of Figure 6A,B show a high correlation for the automatically-derived parameters cumulated leaf area (*R*^2^ = 0.99) and cumulated stem height (*R*^2^ = 0.98) compared to manually-derived parameters. The parameter, cumulated leaf area, shows a linear correlation with a slope of 1.00 and a minimal offset of 326.2 mm^2^. This can be attributed to an inaccurate classification, in particular at the transition of stem and leaf. In contrast, the correlation of the parameter, cumulated stem height, shows an offset of 8.94 mm and a slope of 1.20. We attribute this to fluctuation in the manually-generated reference data, as it was noticed by different operators for the same plant. Although the manual parameterization method has been shown to be reliable [7,39], experimental and unpublished comparisons showed variations of up to 10 mm for the parameter, cumulated stem height, depending on the operator.

The results describe the applicability of surface feature histograms as an important tool for point cloud segmentation with a subsequent parameterization step. The highest rate of inaccurate classification (17.91%) mainly occurred for the stem organs. We claim the high similarity of a leaf that starts to roll out from a cylindrical shape to a rather flat one to be responsible for that. Nevertheless, the presented approach used a repeated least-squares approximation (RANSAC) of a cylinder using only six points. This improved the accuracy of the approximation and the tolerance in the presence of misclassifications.

All plant organs were parameterized automatically and provided reliable values. The approach can be generalized for use in various scenarios with cylindrical objects, e.g., growth analysis of various crops, like maize [14], plant modeling using real 3D images [16] or the derivation of profitable parameters, like stem height from trees [40,41]. The use of surface feature histogram-based classification together with an automated parameterization for leaves and stems has been successfully applied to a barley breeding scenario. Thus, it depicts an important contribution to overcome time-consuming and labor-intensive working steps for data processing as they have been described for the separation of maize and barley organs [7,14]. It constitutes a key skill for phenotyping to meet the requirements of automatization and speed [42,43].

Physiologically, the stem geometry does not show a completely straight shape, as it is necessary for a cylinder approximation. The curved appearance leads to an imprecise parameterization. A division of the stem into smaller cylinders might help to minimize this error. However, the accuracy of a plant stem approximation by a cylinder has not been explored before and has to be further investigated by future work. Nevertheless, the approximation by cylinders is, to date, a *de facto* standard in plant science [14,44].

The effort for measuring the plants with this laser scanning combination rises with the increasing complexity and size of the barley plants. In the early states, a complete plant scan lasts some minutes; in the later states, this rises up to 15 min. The classification time is between 2 min for small point clouds and 6 min for the biggest and most complex point clouds. For the high throughput application, the use of a faster and automated scanning device should be taken into account.

The presented approach shows the automated monitoring of plant growth aiming at a detailed description of the barley organs, leaf and stem. According to the findings of [4], the accuracy of the derived parameters meets the requirements for monitoring growth. Future work will include the improvement of the stem classification, as well as the use of sensor fusion [2,45] as an additional feature for classification.

The use of functional structural plant modeling [46] will help to improve the classification by prior knowledge and to focus on the modeling of the parts of interest, e.g., leaves [47]. As these models provide deformation prediction [16,48], due to extension growth, the prediction accuracy estimation by comparison with scanned time series is possible. This enables the generation of highly accurate and reliable models, describing various types of cereal plants.

We showed an analysis of the two parameters, leaf area and stem height. The derivation of additional parameters, like leaf inclination, stem volume and thickness, or the monitoring of single organs is possible. The latter requires an accurate alignment of the plants in a way that the position of every stem and leaf is nearly stable. Furthermore, a transfer to additional plant species, like cotton [15] or rice [49], has to be tested, as well as the general application independent of the 3D sensor. Low-cost 3D sensors, like the David system [4] or stereo vision [50,51], have been shown to provide 3D images with high resolution and could constitute a cheap and reliable alternative to high-end commercial scanning systems.

The current study is a proof-of-concept for an automated parameterization approach for the complete above-ground plant at the organ level until the end of tillering. The shown drought effects indicate the method's applicability for the detection and monitoring of drought effects on the 3D structure of plants. Nevertheless, to study the effect of drought stress in a biological context, a similar experiment has to be repeated with a bigger set of test plants, more repetitions and accompanying manual measurements.

## 5. Conclusions

The presented approach shows the automated parameter tracking of the organs, leaf and stem, for a barley plant over time. It connects the advantages of surface feature histograms as a description of the laser scanned point cloud with a parametric modeling of the plant organs. A calculation of the stem parameter, cumulated height, is provided, as well as the calculation of the parameter, cumulated leaf area. Surface feature histograms were used to identify the specific organ points from the point cloud. The leaves were parameterized by a triangle meshing, whereas the stems were described by a least-squares cylinder approximation. Its applicability has been realized for the comparison of growth curves of an irrigated and a non-irrigated barley plant within a period of 21 days and can be used for the research of environmental influences on the organ extension growth.

Our approach depicts the automation of the parameterization step to accelerate the analysis of huge amounts of data, as they are typically collected by large-scale phenotyping platforms. The geometrical relationship of the barley surface to further cereal crops suggests that the method can be simply adapted to crops, like rice or wheat. This is the key skill for a deeper understanding of plant extension growth and the link between genotype and the final plant architecture.

## Figures and Tables

**Table 1.** The plant experiment included the monitoring of two plants (irrigated and non-irrigated) at ten measuring dates. To get a more detailed view on the complexity and size of the plant point clouds, the number of leaves and stems are shown, as well as the reached accuracy for the classification of leaf and stem points using the point cloud of the plant of day 21 for training.

	**Date**	**Stem Points**	**Leaf Points**	**Stems**	**Leaves**	**Acc.Leaf**	**Acc. Stem**
		#	#	#	#	%	%
irrigated	0	1,911	14,425	1	3	91.46	92.13
	3	1,667	13,397	1	3	93.36	84.72
	5	2,830	26,625	1	4	95.97	57.49
	7	2,241	20,779	1	4	97.29	91.94
	10	3,475	37,692	2	5	96.78	80.24
	12	5,723	41,244	2	7	96.28	87.55
	14	10,175	57,186	3	8	95.03	88.03
	17	6,743	81,155	3	9	95.54	88.30
	19	13,834	85,159	4	11	94.79	81.13
	21	14,419	125,046	4	15	-	-

non-irrigated	0	2,855	24,782	1	3	92.70	87.83
	3	6,594	31,228	1	3	84.46	62.40
	5	6,710	42,433	1	4	89.75	83.37
	7	7,441	47,604	1	4	89.68	67.46
	10	6,628	81,085	1	6	86.19	89.38
	12	9,442	89,547	2	7	95.71	72.52
	14	13,974	98,555	2	7	93.84	77.74
	17	15,946	115,386	2	7	94.03	85.84
	19	12,209	95,356	2	8	96.88	89.00
	21	20,469	120,199	2	9	94.47	91.24
Ø						93.97	82.09

## References

[1] Hosoi F., Nakabayashi K., Omasa K. (2011). 3-D Modeling of Tomato Canopies Using a High-Resolution Portable Scanning Lidar for Extracting Structural Information. Sensors.

[2] Omasa K., Hosoi F., Konishi A. (2007). 3-D LiDAR imaging for detecting and understanding plant responses and canopy structure. J. Exp. Bot..

[3] Hartmann A., Czauderna T., Hoffmann R., Stein N., Schreiber F. (2011). HTPheno: An image analysis pipeline for high-throughput plant phenotyping. BMC Bioinforma..

[4] Paulus S., Behmann J., Mahlein A.K., Plümer L., Kuhlmann H. (2014). Low-cost 3D systems—Well suited tools for plant phenotyping. Sensors.

[5] Sanz-Cortiella R., Llorens-Calveras J., Escolà A., Arnó-Satorra J., Ribes-Dasi M., Masip-Vilalta J., Camp F., Gràcia-Aguilá F., Solanelles-Batlle F., Planas-DeMartí S. (2011). Innovative LIDAR 3D Dynamic Measurement System to Estimate Fruit-Tree Leaf Area. Sensors.

[6] Van der Heijden G., Song Y., Horgan G., Polder G., Dieleman A., Bink M., Palloix A., van Eeuwijk F., Glasbey C. (2012). SPICY: Towards automated phenotyping of large pepper plants in the greenhouse. Funct. Plant Biol..

[7] Paulus S., Schumann H., Leon J., Kuhlmann H. (2014). A high precision laser scanning system for capturing 3D plant architecture and analysing growth of cereal plants. Biosyst. Eng..

[8] Furbank R., Tester M. (2011). Phenomics-technologies to relieve the phenotyping bottleneck. Trends Plant Sci..

[9] Dhondt S., Wuyts N., Inzé D. (2013). Cell to whole-plant phenotyping: The best is yet to come. Trends Plant Sci..

[10] Wagner B., Santini S., Ingensand H., Gärtner H. (2011). A tool to model 3D coarse-root development with annual resolution. Plant Soil.

[11] Keightley K., Bawden G. (2010). 3D volumetric modeling of grapevine biomass using Tripod LiDAR. Comput. Electron. Agric..

[12] Paulus S., Dupuis J., Mahlein A., Kuhlmann H. (2013). Surface feature based classification of plant organs from 3D laserscanned point clouds for plant phenotyping. BMC Bioinforma..

[13] Munns R., James R., Sirault X., Furbank R., Jones H. (2010). New phenotyping methods for screening wheat and barley for beneficial responses to water deficit. J. Exp. Bot..

[14] Frasson R., Krajewski W. (2010). Three-dimensional digital model of a maize plant. Agric. For. Meteorol..

[15] Paproki A., Sirault X., Berry S., Furbank R., Fripp J. (2012). A novel mesh processing based technique for 3D plant analysis. BMC Plant Biol..

[16] Evers J., Vos J., Yin X., Romero P., van der Putten P., Struik P. (2010). Simulation of wheat growth and development based on organ-level photosynthesis and assimilate allocation. J. Exp. Bot..

[17] Thornley J. (1999). Modelling Stem Height and Diameter Growth in Plants. Ann. Bot..

[18] Rui Y.K., Peng Y.F., Wang Z.R., Shen J.B. (2009). Stem perimeter, height and biomass of maize (Zea mays L.) grown under different N fertilization regimes in Beijing, China. Int. J. Plant Prod..

[19] Araus J.L., Cairns J.E. (2014). Field high-throughput phenotyping: The new crop breeding frontier. Trends Plant Sci..

[20] Fanourakis D., Briese C., Max J.F., Kleinen S., Putz A., Fiorani F., Ulbrich A., Schurr U. (2014). Rapid determination of leaf area and plant height by using light curtain arrays in four species with contrasting shoot architecture. Plant Methods.

[21] Xu L., Henke M., Zhu J., Kurth W., Buck-Sorlin G. (2011). A functional-structural model of rice linking quantitative genetic information with morphological development and physiological processes. Ann. Bot..

[22] Buck-Sorlin G., Hemmerling R., Kniemeyer O., Burema B., Kurth W. (2008). A Rule-Based Model of Barley Morphogenesis, with Special Respect to Shading and Gibberellic Acid Signal Transduction. Ann. Bot..

[23] Bastet J., Müller J., Christen O. LEAFC3-N: Modeling effects of drought stress on photosynthesis, stomatal conductance and transpiration.

[24] Bohmann A., Claus J., Chavarria-Krauser A. Modelling transport processes in tissues and organs at a mesoscopic scale.

[25] Le Gall J., Autret H., Combes D., Renaud C., Barthloot J., Leduc N., Andrieu B., Guérin V., Chelle M., Demotes-Mainard S. Evaluation of a photon tracing model and virtual plants to simulate light distribution within a canopy in a growth chamber.

[26] Vapnik N.V. (1998). Statistical Learning Theory.

[27] Technical Data Perceptron ScanWorks V5 for Romer. http://www.hexagonmetrology.com.ar/en/perceptron-scanworks-v5-for-romer_275.htm#.U6kXKCeZvYM.

[28] Hexagon Metrology GmbH. Technical Data Romer Infinite 2.0. http://www.hexagonmetrology.de/ROMER-Absolute-Arm_860.htm.

[29] Dupuis J., Paulus S., Behmann J., Plümer L., Kuhlmann H. (2014). A Multi-Resolution Approach for an Automated Fusion of Different Low-Cost 3D Sensors. Sensors.

[30] Rusu R., Marton Z., Blodow N., Beetz M. Learning informative point classes for the acquisition of object model maps.

[31] Chang C., Lin C. (2011). LIBSVM : A library for support vector machines. ACM Trans. Intell. Syst. Technol..

[32] Scholkopf B., Smola A.J. (2001). Learning with Kernels: Support Vector Machines, Regularization, Optimization, and Beyond.

[33] Weber C., Hahmann S., Hagen H. Sharp Feature Detection in Point Clouds.

[34] Wiedenbeck M., Züll C., Wolf C., Best H. (2010). Clusteranalyse. Handbuch der Sozialwissenschaftlichen Datenanalyse.

[35] Neimeier W. (2002). Ausgleichungsrechnung.

[36] Pfeifer N., Gorte B., Winterhalder D. Automatic reconstruction of single trees from terrestrial laser scanner data.

[37] Mikhail E., Ackermann F. (1976). Observations and Least Squares.

[38] Fischler M., Bolles R. (1981). Random Sample Consensus: A Paradigm for Model Fitting with Applications to Image Analysis and Automated Cartography. Commun. ACM.

[39] Paulus S., Eichert T., Goldbach H., Kuhlmann H. (2014). Limits of active laser triangulation as an instrument for high precision plant imaging. Sensors.

[40] Liang X., Litkey P., Hyppä J., Kaartinen H., Vastaranta M., Holopainen M. (2012). Automatic Stem Mapping Using Single Scan Terrestrial Laser Scanning. IEEE Trans. Geosci. Remote Sens..

[41] Liang X., Hyppä J. (2013). Automatic Stem Mapping by Merging Several Terrestrial Laser Scans at the Feature and Decision Levels. Sensors.

[42] Houle D., Govindaraju D.R., Omholt S. (2010). Phenomics: The next challenge. Nat. Rev..

[43] Fiorani F., Schurr U. (2013). Future Scenarios for Plant Phenotyping. Ann. Rev. Plant Biol..

[44] Bloomenthal J. Modeling the Mighty Maple.

[45] Berdugo C., Zito R., Paulus S., Mahlein A. (2014). Fusion of sensor data for the detection and differentiation of plant diseases in cucumber. Plant Pathol..

[46] Vos J., Evers J., Buck-Sorlin G., Andrieu B., Chelle M., de Visser P. (2010). Functional-structural plant modeling: A new versatile tool in crop science. J. Exp. Bot..

[47] Uhrmann F., Hügel C., Paris S., Scholz O., Zollhöfer M., Greiner G. A Model-Based Approach to Extract Leaf Features from 3D Scans.

[48] Polani D., Kim J., Martinetz T. (2002). L-System Model of the Vegetative Growth of Winter Barley (Hordeum Vulgare L.). Fifth German Workshop on Artificial Life: Abstracting and Synthesizing the Principles of Living Systems.

[49] Praba M.L., Cairns J.E., Babu R.C., Lafitte H.R. (2009). Identification of physiological traits underlying cultivar differences in drought tolerance in rice and wheat. J. Agron. Crop Sci..

[50] Seitz S., Curless B., Diebel J., Scharstein D., Szeliski R. A Comparison and Evaluation of Multi-View Stereo Reconstruction Algorithms.

[51] Biskup B., Scharr H., Schurr U., Rascher U. (2007). A stereo imaging system for measuring structural parameters of plant canopies. Plant Cell Environ..

